# Investigation of Cardiovascular Health and Risk Factors Among the Diverse and Contemporary Population in London (the TOGETHER Study): Protocol for Linking Longitudinal Medical Records

**DOI:** 10.2196/17548

**Published:** 2020-10-02

**Authors:** Kanika Dharmayat, Maria Woringer, Nikolaos Mastellos, Della Cole, Josip Car, Sumantra Ray, Kamlesh Khunti, Azeem Majeed, Kausik K Ray, Sreenivasa Rao Kondapally Seshasai

**Affiliations:** 1 Department of Primary Care and Public Health Imperial Centre for Cardiovascular Disease Prevention Imperial College London London United Kingdom; 2 Cardiovascular Sciences Research Centre St George's, University of London London United Kingdom; 3 Global eHealth Unit Department of Primary Care and Public Health Imperial College London London United Kingdom; 4 NNEdPro Global Centre for Nutrition and Health in Cambridge University of Cambridge Cambridge United Kingdom; 5 Humanities and Social Science University of Cambridge Cambridge United Kingdom; 6 Primary Care Diabetes and Vascular Medicine University of Leicester Leicester United Kingdom; 7 Department of Primary Care and Public Health Imperial College London London United Kingdom; 8 St George’s Hospital NHS Foundation Trust London United Kingdom

**Keywords:** electronic health records, cardiovascular health, cardiovascular risk factors

## Abstract

**Background:**

Global trends in cardiovascular disease (CVD) exhibit considerable interregional and interethnic differences, which in turn affect long-term CVD risk across diverse populations. An in-depth understanding of the interplay between ethnicity, socioeconomic status, and CVD risk factors and mortality in a contemporaneous population is crucial to informing health policy and resource allocation aimed at mitigating long-term CVD risk. Generating bespoke large-scale and reliable data with sufficient numbers of events is expensive and time-consuming but can be circumvented through utilization and linkage of data routinely collected in electronic health records (EHR).

**Objective:**

We aimed to characterize the burden of CVD risk factors across different ethnicities, age groups, and socioeconomic groups, and study CVD incidence and mortality by EHR linkage in London.

**Methods:**

The proposed study will initially be a cross-sectional observational study unfolding into prospective CVD ascertainment through longitudinal follow-up involving linked data. The government-funded National Health System (NHS) Health Check program provides an opportunity for the systematic collation of CVD risk factors on a large scale. NHS Health Check data on approximately 200,000 individuals will be extracted from consenting general practices across London that use the Egton Medical Information Systems (EMIS) EHR software. Data will be analyzed using appropriate statistical techniques to (1) determine the cross-sectional burden of CVD risk factors and their prospective association with CVD outcomes, (2) validate existing prediction tools in diverse populations, and (3) develop bespoke risk prediction tools across diverse ethnic groups.

**Results:**

Enrollment began in January 2019 and is ongoing with initial results to be published mid-2021.

**Conclusions:**

There is an urgent need for more real-life population health studies based on analyses of routine health data available in EHRs. Findings from our study will help quantify, on a large scale, the contemporaneous burden of CVD risk factors by geography and ethnicity in a large multiethnic urban population. Such detailed understanding (especially interethnic and sociodemographic variations) of the burden of CVD risk and its determinants, including heredity, environment, diet, lifestyle, and socioeconomic factors, in a large population sample, will enable the development of tailored and dynamic (continuously learning from new data) risk prediction tools for diverse ethnic groups, and thereby enable the personalized provision of prevention strategies and care. We anticipate that this systematic approach of linking routinely collected data from EHRs to study CVD can be conducted in other settings as EHRs are being implemented worldwide.

**International Registered Report Identifier (IRRID):**

PRR1-10.2196/17548

## Introduction

### Background

Globally, cardiovascular disease (CVD) is a leading cause of disability and premature mortality, with the number of deaths attributable to CVD rising from 12.3 to 17.3 million between 1990 and 2013 [1]. Furthermore, projections indicate that CVD will remain the leading cause of mortality worldwide for decades [2]. Notwithstanding the public health importance of CVD and its risk factors, the use of proven preventative strategies to mitigate risk is far from satisfactory due to a multitude of factors including complex intra- and interregional distribution of the determinants of CVD [1,3]. For instance, deaths attributable to different types of CVD vary between China and the United Kingdom. This variation may, in part, reflect the prevalence of different underlying risk factors such as hypertension and diabetes [4]. Thus, with regions around the world undergoing major epidemiological transitions, a population strategy aimed at reducing the burden of classical risk factors can have a major impact on CVD risk reduction [5].

### Population Prevention Strategy in England

In England, the National Health Service (NHS) established a population-based Health Check program in 2009 to provide a systematic assessment and management of CVD risk for adults. Individuals aged 40-74 years without pre-existing CVD, diabetes, or hypertension are invited for a five-yearly check-up to identify and to receive advice and treatment for their risk of having a CVD event [6]. Commissioned by local authorities and delivered primarily through primary care practices, henceforth referred to as general practices (GP), this program aims to prevent around 2000 deaths and 9500 nonfatal myocardial infarctions (MI) and strokes annually [7,8]. As a free service for all eligible individuals, this program has the potential to reduce health inequalities associated with CVD [7,8], assuming equitable uptake and universal coverage. Available data suggest that between 2011-12, NHS Health Check coverage in east London was quite high at 73% (compared to national coverage of 30%). Of those that attended the screening, over a third belonged to ethnic minority groups such as south Asians and Black African/Caribbeans [9-13]. While modification of individual risk factors (eg, blood pressure) has generally been shown to be associated with improved health outcomes (eg, lower incidence of ischaemic stroke), favorable changes in risk factor distribution at a societal level have also contributed to overall improvements in community health [14]. For example, in 1989, in Poland, the increased accessibility and consumption of fruits and vegetables through the opening of markets resulted in a reduction in CVD mortality [15]. By contrast, Japanese migrants, who adopted the diet and lifestyle of the United States (US) population, relative to those who retained their original dietary patterns, had a higher prevalence of CVD [16]. Given the variation in ethnic-specific diet and lifestyle habits, and given genetic differences across population groups around the world, there is currently an unmet need to understand the complex relationship between ethnicity and factors as diverse as heredity, environment, diet, lifestyle, and socioeconomic factors (and their mutual interaction) in determining the burden, distribution, and temporal trends in both CVD risk factors and clinical CVD outcomes and cause-specific death.

Dietary exposures determine nutritional status, which may, in turn, impact CVD outcomes. As nutritional status is a key intermediary in the development of CVD, health services need to measure nutritional status as well as associated dietary exposures in order to guide appropriate preventative measures, particularly in multiethnic populations in primary care. The National Diet and Nutrition Survey (NDNS) and components of the National Survey of Health and Development (NSHD) have been developed to assess dietary exposure patterns, nutritional status, and associated social as well as biological factors within a sample of UK populations [17-19]. However, to date, no systematic attempts have been made to harness these data to boost or complement information gleaned from population-based studies investigating ethnic-specific CVD risk factor burden or risk [20].

### Calculation of CVD Risk

The risk of CVD in an asymptomatic individual is based on risk factor data collected by their primary care physician to produce a numerical estimate. Traditional risk scores, such as those derived from the Framingham risk equation, have potential limitations, primarily because they were derived from a cohort of predominantly White individuals in the United States [21,22]. Moreover, they do not make allowances for factors such as socioeconomic status, resulting in systematic over- or underestimation of CVD risk based on the population studied [21,22]. With the emergence of obesity and diabetes as key global cardiovascular risk factors [22], the QRISK2 CVD risk calculator was developed and calibrated, enabling adjustments for more contemporary risk factors in the United Kingdom [22,23]. Despite these considerations, the representation of ethnic minorities in the cohort used to derive QRISK2 was approximately 1% for South Asians and 0.5% for Afro-Caribbean individuals, with the likelihood of underestimation of 10-year CVD event risk in these population groups [21].

Furthermore, QRISK prediction models contain imputations due to missing data. While established statistical methods are often used, considerations such as imputing cholesterol values to individuals without vascular disease could lead to weakened associations between cholesterol and CVD [24], as was observed in the first iteration of QRISK where about 80% of cholesterol measures were imputed. Hence, additional validation of QRISK2 in a contemporaneous multiethnic population with more systematic approaches to the collation of exposures than previously possible would be beneficial [21].

These systems need to be interoperable to enable a systematic analysis of routine healthcare data collected in electronic health records (EHRs). However, over 100 commercial EHR vendors are operating within the NHS across primary, secondary, and tertiary sectors [25]. While organizations are beginning to link data from different EHR systems, there is still fragmentation and absence of vital data as, for example, GPs serving multiethnic populations are not providing their data. Moreover, given that establishing bespoke prospective studies on CVD prevention can be prohibitively expensive, using real-life routinely collected data from EHRs and subsequent linkage of these datasets across different sectors can provide invaluable systematic approaches to conducting research.

We developed the following study specific objectives:

1. To robustly quantify and characterize, across diverse ethnic groups and sociodemographic groups, the burden of CVD risk factors among NHS Health Check attendees in a diverse population in London.
2. To prospectively study the incidence of CVD within this population by EHR linkage.
3. To validate existing CVD risk prediction algorithms and to develop novel, bespoke algorithms for CVD risk prediction among diverse multiethnic population groups.
4. To characterize dietary exposures and nutritional status as well as their associations with CVD outcomes, using data from index assessment methods that mirror or correlate well with those used in national surveillance.

## Methods

### Study Design and Setting

**Figure 1:**
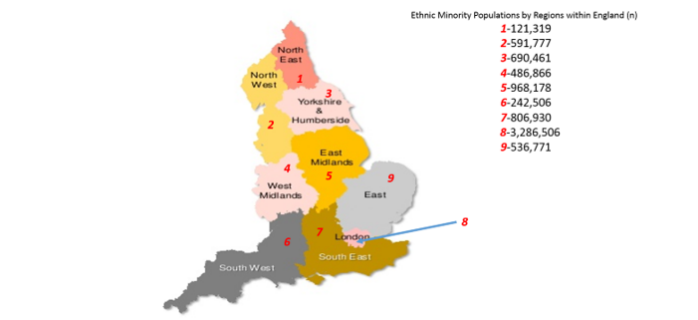
Data from 2011, showing ethnic minority populations across the regions of England [26].

**Figure 2:**
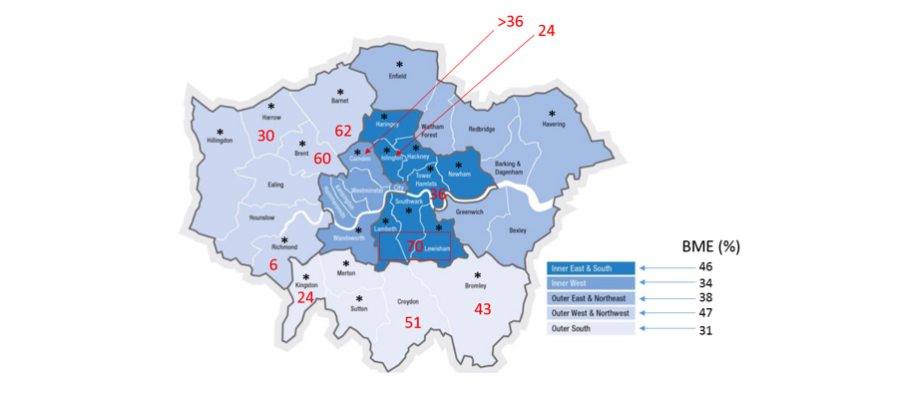
Map of London showing the number of GP practices utilizing the EMIS system (red), boroughs utilizing EMIS (asterisk), and proportion of Black and Minority Ethnic (BME) by region [26-36].

We propose to conduct a large-scale, cross-sectional observational study of NHS Health Check attendees in London with additional virtual longitudinal follow-up established via electronic health record linkage of their primary (NHS Health Check) and secondary care (hospital) data over 10 years starting in 2009. One of the major providers of EHR software to GP practices is Egton Medical Information Systems (EMIS). GPs utilizing this EHR will be eligible to participate. Figure 1 illustrates the number of individuals belonging to ethnic minority groups within London. Such a high prevalence of diverse ethnic groups in London, relative to other areas in England, coupled with high utilization of the EMIS system (Figure 2), lends itself naturally to London as the ideal backdrop for our proposed investigation.

### Recruitment

GP practices using EMIS Web clinical systems across London will be invited to provide practice-wide consent to participating in the study by way of sharing pseudonymized data on individuals undergoing NHS Health Checks within their practices between 2009 and 2018. GP practices across London are being informed of the study and recruited through liaising with clinical research networks through information letters and Clinical Commissioning Group meetings. Data will be remotely extracted centrally by EMIS using Read codes.

### Data Collection and Linkage

Our study, designed as a population-based registry at the outset, will collect, alongside NHS Health Check data, additional demographic variables and prescription information to help build a large and robust repository of primary care data on residents of London undergoing NHS Health Check from 2009 onwards in GP practices.

All individuals seeking healthcare in the United Kingdom are assigned a unique identifier (ie, their NHS number). This number allows the allocation of any subsequent data collected to the individual’s record and enables linkage with diverse data sources. For instance, the Office for National Statistics and Hospital Episode Statistics databases maintain records on mortality and clinical outcomes of individuals utilizing secondary healthcare services, respectively. Data from these sources can be linked through NHS numbers to the NHS Health Check data to provide a comprehensive and contemporary database on CVD risk factors (predictor variables) and outcomes across different ethnic groups. Other data sources recording clinical CVD outcomes such as the Myocardial Infarction National Audit Project and the National Institute of Cardiovascular Outcomes Research will also be harnessed to provide additional details relevant to this study.

This process of data sharing and linking, while protecting an individual’s confidentiality, is possible due to pseudonymization and will be conducted by EMIS and a third party organization familiar with bespoke linkage. Using Open Pseudonymiser Software, EMIS will create a pseudonymized version of NHS numbers for individuals who meet the inclusion criteria. The data will be encrypted with a project-specific key. By using this encrypted key, an external organization will be able to link the NHS Health Check data with outcomes data through a similar pseudonymization process and a Global Unique Identifier (GUID). The research team will only have access to completely linked anonymized data as the linkage of NHS Health Check data with outcomes data will not contain the GUID.

### Sample Size

Although data are not available for 2009-2011, of the invited population for NHS Health Check in 2016-17, 51.6% and 49.9% were residing in London and the rest of England, respectively [27]. In 2015, EMIS utilization in GP clinics was approximately 55%. Therefore, if 50% of all NHS Health Check attendees visit GP clinics utilizing the EMIS system, then it can be assumed that 587,273 individuals in London are eligible for consideration in this study between 2011 and 2017 [28,29]. Due to a lack of visibility of the true number of GP practices using a particular system, Figure 2 highlights that the majority of the areas in London do utilize the EMIS system, and, therefore, we anticipate that the intended sample size will be met.

To develop novel risk prediction tools for population groups, we anticipate that data will be available on approximately 200,000 individuals across London between 2011 and 2017. During 2009-13, of those with a recorded ethnic group, uptake of the NHS Health Check across England was greatest amongst South Asians (19.2%), Black Caribbeans (19.6%), Black Africans (15.7%), and Chinese (15.3%). Attendance was 16.9% in all other recorded ethnic groups and 17.4% in white Caucasians. Extrapolating the current rate of CVD incidence (2.4%) for people aged below 79 years in England on our estimated study sample of 200,000, we expect to encounter 15,000 CVD events giving 100% power to observe relative risks as modest as 1.2-1.5 [23]. Within population subgroups, the power to detect overall association is expected to range from 49% to 100% [38,39]. Given the large Black and minority ethnic (BME) population in London and considering the above numbers, we estimate that our study sample will be highly representative of a diverse cohort [38,39].

### Predictor and Outcome Variables

Cardiovascular risk factors (predictor variables) used to calculate QRISK2 include age, sex, ethnicity, index of multiple deprivations, smoking status, diabetes status, family history of CVD, chronic kidney disease (stage 4 or 5), atrial fibrillation, hypertension treatment, rheumatoid arthritis, lipids, blood pressure, and BMI. EMIS will also extract prescription data. CVD outcomes will include fatal and nonfatal coronary heart disease, fatal and nonfatal stroke, heart failure, all CVD-related hospitalizations (including those for revascularization), and all-cause mortality.

### Harmonization and Cleaning of Data

Since the data for our study will be extracted at a single source (EMIS), this is likely to obviate the need for harmonizing, although we will undertake detailed quality checks (both at baseline and on follow-up) and undertake data cleaning, where necessary.

### Statistical Analyses

By using appropriate statistical tests to perform univariate comparisons, the collected data will be initially analyzed to explore the cross-sectional characteristics of this population. Skewed data will be transformed to an approximately normal distribution, with the calculation of Pearson correlation coefficients and partial correlation coefficients for relevant factors of interest.

Cross-sectional associations of baseline risk factors will be studied with adjustments for appropriate confounders using logistic regression analyses. Prospective associations between individual risk factors and CVD outcomes will be evaluated using Kaplan-Meier survival curves and further quantified by fitting Cox proportional hazards regression models with multivariable adjustments. Where possible, associations will be studied after making allowances for the time-varying effects of both exposures and potential confounders. Detailed analyses of the associations will be conducted in prespecified subgroups of participants (including, but not restricted to, age, sex, conventional risk factors, and ethnicity). Established methods of calibration (Hosmer-Lemeshow), discrimination (C or D statistic), and reclassification (net reclassification index, integrated discrimination improvement) will be used to study the predictive ability of individual risk factors on CVD outcomes, with prespecified analyses involving multiple ethnic groups to enable direct, head-to-head comparisons of any differences.

Additional analyses will be conducted to quantify the impact of factors such as access to healthcare and utilization rates (of NHS Health Checks), prescription rates and medication compliance, and patterns of healthy behavior on CVD risk (both overall and, where possible, within subgroups). Standardized rates of prescription drug use will be calculated to investigate the extent of correlation between the present use of cardiovascular medication and cardiovascular risk. Variations in prescription patterns of CVD prevention and risk factor control, discontinuation of prescribed medication(s), and adverse effects to prescription medications from various therapeutic categories such as anti-hypertensive agents, lipid-lowering drugs (statins), and antidiabetic medication will be further assessed for their impact on CVD as time-dependent covariates in Cox regression models. Physical measurements within the NHS Health Check program will be obtained by the GP practices according to standard operating procedures, anticipating minimum inter-observer variability. Analyses to explore potential sources of heterogeneity concerning physical measurements (besides other characteristics) will also be conducted.

New and existing risk prediction models will be compared in terms of their ability to predict the onset of CVD across diverse population groups to develop improved risk prediction tools for primary prevention of CVD using outcomes data up to 2020. The resulting CVD risk algorithm will be calibrated by further analyses of CVD outcomes data of these patients through to 2027.

## Results

The study is ongoing with enrollment underway since January 2019. Recruitment ended in January 2020, with extraction and linkage completed by March 2020. We expect the initial results for NHS Checks conducted between 2009 and 2018 and linked to Hospital Episode Statistics and Office for National Statistics to be published in mid-2021. This research has been supported by an unrestricted investigator-initiated research grant from Regeneron Pharmaceuticals to Imperial College London in 2015.

### Ethical Considerations

The study protocol underwent an external peer review before the ethics submission. This study has received a favorable ethics opinion by the East Scotland Research Ethics Committee (Ref: 17-ES-0104), Health Research Authority, and adopted by the clinical research network portfolio in November 2018.

## Discussion

Nationally delivered, the NHS Health Check program has the potential for universal outreach in the United Kingdom. However, differences in uptake of this program may lead to inadvertent and undesirable health inequalities (for instance, those already more likely to engage with the health care benefiting from increased contact while many who are less likely to engage with healthcare not availing themselves of this opportunity). However, it is unclear whether (and to what extent) ethnic differences at a population level drive such disparities, and whether targeting specific sociodemographic subgroups within the population might help mitigate CVD risk.

The complex interplay and variations of individual risk factors with wider determinants between and within regions and population groups reinforce the need for a detailed understanding of different cultural, socioeconomic, and epidemiological contexts for developing tailored population strategies.

Given its ethnic diversity (and a population of 8 million representing around 270 nationalities), London provides the ideal backdrop for studying CVD risk factor burden in all the permutations mentioned above. It has been estimated that the proportion of BME groups in London is 41% (10% in the rest of England). Moreover, a report commissioned by the King’s Fund found greater health inequalities in London than in the rest of England [13]. For instance, although CVD death rates are reported to be higher in the North than in the South of England, 10 of the 33 London boroughs are currently experiencing above-national-average mortality [26-28,30-37]. These factors highlight the need for a more in-depth understanding of the determinants of CVD risk based on ethnicity and other key demographic variables, which could then pave the way for more effective societal interventions.

Thus, the aim of our study, using NHS Health Check data in London, is to understand the burden of CVD risk factors and to eventually validate/develop ethnic-specific CVD risk prediction tools to inform public health policy and guidelines which could lead to improvements in overall health outcomes and reduction in healthcare costs.

Ethnic diversity in England in general, and London in particular, has hitherto not been systematically captured in large-scale studies creating uncertainty in the existing guidelines on CVD risk prevention. QRISK2 score has been shown to underpredict CVD risk in population groups such as South Asians, in part reflecting low representation of the ethnic groups in the cohorts to derive sufficient information to inform risk prediction tools adequately [40,41]. This disparity may be attributed to the low participation of GP practices in London who, based on the location of these populations, are likely to treat the greatest number of BME groups relative to the rest of England. Thus, in certain ethnic groups, there may be a missed opportunity to mitigate CVD risk as they are at greater risk of CVD but have a systematic under-estimation/-representation of risk. Given that EHR data in many GP practices within London have not been utilized for investigating CVD across the multiethnic population they serve, there is a unique opportunity to recruit these practices to share their EHR data and link with other EHR sources to create a registry of varied population groups.

Moreover, the data from this study could help develop bespoke risk prediction models across diverse ethnic groups that can be embedded into the existing GP EHR systems. Following the development of such models, individuals of diverse ethnicities could potentially be reclassified into higher- or lower-than-predicted CVD risk categories, thereby enabling more tailored approaches to prevention or intervention. By contrast, if conventional risk factors were found not to be associated with discernible differences in CVD risk across various ethnic groups, then our findings will have the potential to influence future research aimed at addressing alternative (and hitherto unexplored) risk factors (such as genetic variation or dietary factors) contributing to excess CVD risk in ethnic minorities. Further offshoots from the TOGETHER research program will not only be able to address some of the novel hypotheses regarding ethnic variation in CVD risk but also serve as a platform for pragmatic randomized controlled trials to explore, for example, various emerging health technologies. One such approach could be leveraging the use of mobile phones/smartphones and applications (apps) to modify risk factors [41] and then identify the key elements to effective and sustainable approaches for reducing the risk of CVD [42]. Furthermore, there could be an opportunity to leverage the derived dataset to perform machine learning techniques, which could be potentially incorporated into creating digital health interventions [43].

As CVD burden varies between population groups and by regions, our study has the potential to investigate the expression of this disease and the contemporary risk factors in a large multiethnic cohort. Thus, our research can be considered a contemporary Framingham study on a large scale. Combining a real-life observational approach with the potential for yearly updates from the EHRs will offer new insights and understanding of the burden of risk factors for re-calibration of the variables used to derive risk prediction tools and dynamic improvement of risk prediction algorithms. Longitudinal follow-up will help quantify the incidence of CVD and, therefore, the derivation of population attributable risk (PAR) [44]. The ability to predict the impact of removing a particular risk factor in specific population groups and the risk of developing subsequent CVD is imperative for service provision and evaluating current public health strategies for CVD risk reduction. Thus, given its sample size, our study will have sufficient power to investigate CVD risk factor burden across diverse ethnicities among NHS Health Check attendees enabling further refinement of guidelines and will therefore have the potential to improve the overall public health of England in the foreseeable future.

The proposed analyses are in keeping with statistical analyses conducted in other similar large-scale population-based studies [45-47]. As the NHS Health Check program collects simple lifestyle-related data, a proportion of the population from this study will be investigated using the NDNS database to derive a more detailed understanding of the impact of nutritional determinants on CVD [48-50]. As this study aims to provide current estimates of CVD risk factor burden in a very large sample of ethnically diverse individuals in London (and following successful linkage with outcomes databases, estimates of association), we believe that this project surpasses every other contemporary study in terms of both its scale as well as representativeness (from an ethnic perspective). While our estimates of association and risk prediction derived from this cohort of CVD-free individuals (at baseline) may be similar to those already obtained from studies conducted in predominantly Western populations, these estimates will nevertheless be significantly refined across diverse ethnicities given the power and precision afforded by the study.

Despite the development of evidence-based guidelines and tools by the NHS, opportunities to reduce CVD mortality and morbidity are still being missed. Premature mortality and high healthcare costs associated with CVD underscore the need for a more detailed investigation into CVD risk across multiple ethnic groups since residual CVD risk remains a major challenge for health systems around the world. With the identification of more than 200 risk factors [51], determining the specific risk factors in population groups is imperative. Therefore, with this study examining an ethnically diverse and contemporary population in London, the findings will have the potential to be applied to other (hitherto under-investigated) ethnicities, and towns and cities around the world that have considerable population diversity. Finally, the systematic approach of linking data from different sources can be utilized by other health systems that have implemented EHRs.

## Abbreviations

BME: Black and Minority Ethnic
CVD: cardiovascular disease
EMIS: Egton Medical Information Systems
GP: general practice
GUID: global unique identifier
MI: myocardial infarction
NDNS: National Diet and Nutrition Survey
NHS: National Health Service
NSHD: National Survey of Health and Development

## References

[1] Roth Gregory A, Huffman Mark D, Moran Andrew E, Feigin Valery, Mensah George A, Naghavi Mohsen, Murray Christopher J L (2015). Global and regional patterns in cardiovascular mortality from 1990 to 2013. Circulation.

[2] Kelly B, Fuster V (2010). Promoting cardiovascular health in the developing world a critical challenge to achieve global health. editors.

[3] Yusuf Salim, Wood David, Ralston Johanna, Reddy K Srinath (2015). The World Heart Federation's vision for worldwide cardiovascular disease prevention. Lancet.

[4] Neal B, Chapman N, Patel A (2002). Managing the global burden of cardiovascular disease. European Heart Journal Supplements.

[5] Deaton C, Froelicher ES, Wu LH, Ho C, Shishani K, Jaarsma T (2011). The global burden of cardiovascular disease. Eur J of Cardiovasc Nurs.

[6] Dalton Andrew R H, Bottle Alex, Okoro Cyprian, Majeed Azeem, Millett Christopher (2011). Uptake of the NHS Health Checks programme in a deprived, culturally diverse setting: cross-sectional study. J Public Health (Oxf).

[7] Robson John, Dostal Isabel, Sheikh Aziz, Eldridge Sandra, Madurasinghe Vichithranie, Griffiths Chris, Coupland Carol, Hippisley-Cox Julia (2016). The NHS Health Check in England: an evaluation of the first 4 years. BMJ Open.

[8] Forster Alice S, Burgess Caroline, McDermott Lisa, Wright Alison J, Dodhia Hiten, Conner Mark, Miller Jane, Rudisill Caroline, Cornelius Victoria, Gulliford Martin C (2014). Enhanced invitation methods to increase uptake of NHS health checks: study protocol for a randomized controlled trial. Trials.

[9] Robson John, Dostal Isabel, Madurasinghe Vichithranie, Sheikh Aziz, Hull Sally, Boomla Kambiz, Griffiths Chris, Eldridge Sandra (2017). NHS Health Check comorbidity and management: an observational matched study in primary care. Br J Gen Pract.

[10] Chang Kiara Chu-Mei, Soljak Michael, Lee John Tayu, Woringer Maria, Johnston Desmond, Khunti Kamlesh, Majeed Azeem, Millett Christopher (2015). Coverage of a national cardiovascular risk assessment and management programme (NHS Health Check): Retrospective database study. Prev Med.

[11] Asaria Perviz, Fortunato Lea, Fecht Daniela, Tzoulaki Ioanna, Abellan Juan Jose, Hambly Peter, de Hoogh Kees, Ezzati Majid, Elliott Paul (2012). Trends and inequalities in cardiovascular disease mortality across 7932 English electoral wards, 1982-2006: Bayesian spatial analysis. Int J Epidemiol.

[12] Artac M, Dalton A R H, Babu H, Bates S, Millett C, Majeed A (2013). Primary care and population factors associated with NHS Health Check coverage: a national cross-sectional study. J Public Health (Oxf).

[13] Raleigh V, Tian Y, Goodwin N, Dixon A, Thompson J, Millett C, Soljak M General practice in London Supporting improvements in quality. London: The King's Fund.

[14] Chow Clara Kayei, Lock Karen, Teo Koon, Subramanian S V, McKee Martin, Yusuf Salim (2009). Environmental and societal influences acting on cardiovascular risk factors and disease at a population level: a review. Int J Epidemiol.

[15] Zatonski W A, McMichael A J, Powles J W (1998). Ecological study of reasons for sharp decline in mortality from ischaemic heart disease in Poland since 1991. BMJ.

[16] Marmot M G, Syme S L (1976). Acculturation and coronary heart disease in Japanese-Americans. Am J Epidemiol.

[17] Wells Jonathan C, Sawaya Ana Lydia, Wibaek Rasmus, Mwangome Martha, Poullas Marios S, Yajnik Chittaranjan S, Demaio Alessandro (2020). The double burden of malnutrition: aetiological pathways and consequences for health. Lancet.

[18] Frayn K, Stanner Sara, Coe Sarah (2018). Diet, Nutrition and Emerging Risk Factors. Diet, Nutrition and Emerging Risk Factors.

[19] Maddock Jane, Ziauddeen Nida, Ambrosini Gina L, Wong Andrew, Hardy Rebecca, Ray Sumantra (2018). Adherence to a Dietary Approaches to Stop Hypertension (DASH)-type diet over the life course and associated vascular function: a study based on the MRC 1946 British birth cohort. Br J Nutr.

[20] Public HE (2009). National Diet and Nutrition Survey. Years 1 to 9 of the Rolling Programme (2008/.

[21] Tillin Therese, Hughes Alun D, Whincup Peter, Mayet Jamil, Sattar Naveed, McKeigue Paul M, Chaturvedi Nish, SABRE Study Group (2014). Ethnicity and prediction of cardiovascular disease: performance of QRISK2 and Framingham scores in a U.K. tri-ethnic prospective cohort study (SABRE--Southall And Brent REvisited). Heart.

[22] Hippisley-Cox Julia, Coupland Carol, Vinogradova Yana, Robson John, Minhas Rubin, Sheikh Aziz, Brindle Peter (2008). Predicting cardiovascular risk in England and Wales: prospective derivation and validation of QRISK2. BMJ.

[23] Volmink J A, Newton J N, Hicks N R, Sleight P, Fowler G H, Neil H A (1998). Coronary event and case fatality rates in an English population: results of the Oxford myocardial infarction incidence study. The Oxford Myocardial Infarction Incidence Study Group. Heart.

[24] Hippisley-Cox Julia, Coupland Carol, Vinogradova Yana, Robson John, May Margaret, Brindle Peter (2007). Derivation and validation of QRISK, a new cardiovascular disease risk score for the United Kingdom: prospective open cohort study. BMJ.

[25] Parliamentary Office of Science and Technology.

[26] Public Health England Profiles Fingertips. phe.

[27] Trust for London 2018.

[28] Lewisham Clinical Commissioning Group.

[29] Public Health England Fingertips phe. org.

[30] Trust for London (2019). London's geography and population.

[31] Public Health England Profiles Fingertips. phe.

[32] Healthwatch Camden (2019). GP practices in Camden: a study of variation 2015. http://www.healthwatchcamden.co.uk/sites/default/files/gp_report_-_final_december_2015_distributed.pdf.

[33] Barnet Clinical Commissioning Group.

[34] (2019). Richmond Clinical Commissioning Group.

[35] London Borough of Bromley.

[36] (2019). Digital Healthcare Strategy 2015-2020. Croydon Clinical Commissioning Group.

[37] Croydon Clinical Commissioning Group.

[38] Di Angelantonio Emanuele, Sarwar Nadeem, Perry Philip, Kaptoge Stephen, Ray Kausik K, Thompson Alexander, Wood Angela M, Lewington Sarah, Sattar Naveed, Packard Chris J, Collins Rory, Thompson Simon G, Danesh John, Emerging Risk Factors Collaboration (2009). Major lipids, apolipoproteins, and risk of vascular disease. JAMA.

[39] Danesh J, Wheeler JG, Hirschfield GM (2004). C-reactive protein and other circulating markers of inflammation in the prediction of coronary heart disease. N Engl J Med.

[40] George Julie, Mathur Rohini, Shah Anoop Dinesh, Pujades-Rodriguez Mar, Denaxas Spiros, Smeeth Liam, Timmis Adam, Hemingway Harry (2017). Ethnicity and the first diagnosis of a wide range of cardiovascular diseases: Associations in a linked electronic health record cohort of 1 million patients. PLoS One.

[41] Woringer M (2019). , Dharmayat, K., Greenfield, G., Bottle, A. Ray, K.

[42] Volker N (2014). , Davey, R., Cochrane, T., Williams, L. Clancy, T.

[43] Triantafyllidis Andreas K, Tsanas Athanasios (2019). Applications of Machine Learning in Real-Life Digital Health Interventions: Review of the Literature. J Med Internet Res.

[44] Macera CA, Powell KE (2001). Population attributable risk: implications of physical activity dose. Med Sci Sports Exerc.

[45] Langer Robert D, White Emily, Lewis Cora E, Kotchen Jane M, Hendrix Susan L, Trevisan Maurizio (2003). The Women's Health Initiative Observational Study: baseline characteristics of participants and reliability of baseline measures. Ann Epidemiol.

[46] Anderson G, Cummings S, Freedman L (1998). Design of the Women's Health Initiative clinical trial and observational study. Control Clin Trials.

[47] Danesh J, Erqou S, Walker M, Thompson S G, Tipping R, Ford C, Pressel S, Walldius G, Jungner I, Folsom A R, Chambless L E, Knuiman M, Whincup P H, Wannamethee S G, Morris R W, Willeit J, Kiechl S, Santer P, Mayr A, Wald N, Ebrahim S, Lawlor D A, Yarnell J W G, Gallacher J, Casiglia E, Tikhonoff V, Nietert P J, Sutherland S E, Bachman D L, Keil J E, Cushman M, Psaty B M, Tracy R P, Tybjaerg-Hansen A, Nordestgaard B G, Frikke-Schmidt R, Giampaoli S, Palmieri L, Panico S, Vanuzzo D, Pilotto L, Simons L, McCallum J, Friedlander Y, Fowkes F G R, Lee A J, Smith F B, Taylor J, Guralnik J, Phillips C, Wallace R, Blazer D, Khaw K T, Jansson J H, Donfrancesco C, Salomaa V, Harald K, Jousilahti P, Vartiainen E, Woodward M, D'Agostino R B, Wolf P A, Vasan R S, Pencina M J, Bladbjerg E M, Jorgensen T, Moller L, Jespersen J, Dankner R, Chetrit A, Lubin F, Rosengren A, Wilhelmsen L, Lappas G, Eriksson H, Bjorkelund C, Cremer P, Nagel D, Tilvis R, Strandberg T, Rodriguez B, Bouter L M, Heine R J, Dekker J M, Nijpels G, Stehouwer C D A, Rimm E, Pai J, Sato S, Iso H, Kitamura A, Noda H, Goldbourt U, Salomaa V, Salonen J T, Nyyssönen K, Tuomainen T-P, Deeg D, Poppelaars J L, Meade T, Cooper J, Hedblad B, Berglund G, Engstrom G, Döring A, Koenig W, Meisinger C, Mraz W, Kuller L, Selmer R, Tverdal A, Nystad W, Gillum R, Mussolino M, Hankinson S, Manson J, De Stavola B, Knottenbelt C, Cooper J A, Bauer K A, Rosenberg R D, Sato S, Naito Y, Holme I, Nakagawa H, Miura H, Ducimetiere P, Jouven X, Crespo C, Garcia-Palmieri M, Amouyel P, Arveiler D, Evans A, Ferrieres J, Schulte H, Assmann G, Shepherd J, Packard C, Sattar N, Cantin B, Lamarche B, Després J-P, Dagenais G R, Barrett-Connor E, Wingard D, Bettencourt R, Gudnason V, Aspelund T, Sigurdsson G, Thorsson B, Trevisan M, Witteman J, Kardys I, Breteler M, Hofman A, Tunstall-Pedoe H, Tavendale R, Lowe G D O, Ben-Shlomo Y, Howard B V, Zhang Y, Best L, Umans J, Onat A, Meade T W, Njolstad I, Mathiesen E, Lochen M L, Wilsgaard T, Gaziano J M, Stampfer M, Ridker P, Ulmer H, Diem G, Concin H, Rodeghiero F, Tosetto A, Brunner E, Shipley M, Buring J, Cobbe S M, Ford I, Robertson M, He Y, Ibanez A M, Feskens E J M, Kromhout D, Collins R, Di Angelantonio E, Kaptoge S, Lewington S, Orfei L, Pennells L, Perry P, Ray K, Sarwar N, Scherman M, Thompson A, Watson S, Wensley F, White I R, Wood A M, Emerging Risk Factors Collaboration (2007). The Emerging Risk Factors Collaboration: analysis of individual data on lipid, inflammatory and other markers in over 1.1 million participants in 104 prospective studies of cardiovascular diseases. Eur J Epidemiol.

[48] Almoosawi S, Cole D, Nicholson S (2014). Biomarkers of diabetes risk in the National Diet and Nutrition Survey rolling programme (2008?2011). J Epidemiol Community Health.

[49] Ashwell Margaret, Gibson Sigrid (2009). Waist to height ratio is a simple and effective obesity screening tool for cardiovascular risk factors: Analysis of data from the British National Diet And Nutrition Survey of adults aged 19-64 years. Obes Facts.

[50] Woodside Jayne V, Young Ian S, McKinley Michelle C (2013). Fruit and vegetable intake and risk of cardiovascular disease. Proc Nutr Soc.

[51] Hobbs F D R (2004). Cardiovascular disease: different strategies for primary and secondary prevention?. Heart.

